# Identification of volatile components from oviposition and non-oviposition plants of *Gasterophilus pecorum* (Diptera: Gasterophilidae)

**DOI:** 10.1038/s41598-020-72378-9

**Published:** 2020-09-25

**Authors:** Ran Zhou, Ke Zhang, Tiange Zhang, Tong Zhou, Hongjun Chu, Yan Ge, Chen Wang, Kai Li

**Affiliations:** 1 Key Laboratory of Non-Invasive Research Technology for Endangered Species, School of Ecology and Nature Conservation, Beijing Forestry University, Beijing, 100083 China; 2 Wildlife Conservation Office of Altay Prefecture, Altay, 836599 Xinjiang China

**Keywords:** Chemical ecology, Metabolomics, Conservation biology, Plant physiology, Secondary metabolism, Entomology

## Abstract

Oviposition by *Gasterophilus pecorum* on shoot tips of *Stipa caucasica* is a key determinant of its severe infection of the reintroduced Przewalski’s horse (*Equus przewalskii*). Volatiles in shoots of grasses on which Przewalski’s horse feeds, including *S*. *caucasica* at preoviposition, oviposition, and postoviposition stages of *G. pecorum*, *S. caucasica*, *Stipa orientalis*, and *Ceratoides latens* at the oviposition stage, and *S. caucasica* in various growth periods, were collected by dynamic headspace adsorption and analyzed by automatic thermal desorption gas chromatography-mass spectrometry. Among five volatiles with highest relative contents under three sets of conditions, caprolactam and 3-hexen-1-ol,(Z)- were common to all samples. Caprolactam was highest in *C. latens* at oviposition stage of *G. pecorum* and lowest in *S. caucasica* at postoviposition stage, and that of 3-hexen-1-ol,(*Z*)- was lowest in *C. latens* and highest in *S. caucasica* at its oviposition stage. Particularly, in *S. caucasica* during the three oviposition phenological stages of *G*. *pecorum*, 3-hexen-1-ol,acetate,(Z)-, 2(5H)-furanone,5-ethyl-, and 3-hexen-1-ol,acetate,(*E*)- were unique, respectively, to the preoviposition, oviposition, and postoviposition stages; in three plant species during the oviposition stage of *G. pecorum*, 3-hexen-1-ol,acetate,(Z)-, 3-hexenal, and 1-hexanol were unique to *S. orientalis*, acetic acid, hexanal, and 2(5H)-furanone,5-ethyl- to *S. caucasica*, and 1,3,6-octatriene,3,7-dimethyl-, *cis*-3-hexenyl isovalerate, and acetic acid hexyl ester to *C. latens*; in *S. caucasica*, 2-undecanone,6,10-dimethyl- was unique to the early growth period, acetic acid and 2(5H)-furanone,5-ethyl- to the flourishing growth period, and 3-hexen-1-ol,acetate,(*Z*)- and 1,3,6-octatriene,3,7-dimethyl- to the late growth period. Furthermore, substances specific to *S. orientalis* and *C. latens* were also present in *S. caucasica*, except at oviposition stage. Our findings will facilitate studies on *G. pecorum*’s adaptation to the arid desert steppe and its future control.

## Introduction

After Przewalski’s horse (*Equus przewalskii*), an endangered species, was reintroduced to its country of origin at Kalamaili Nature Reserve (KNR) for ungulates in Xinjiang, infection by *Gasterophilus* spp. became an increasingly serious issue. The infection intensity of *Gasterophilus* spp. is higher in Przewalski’s horses than in Mongolian wild asses (*Equus hemionus*) within the same area. The infection rate by *Gasterophilus pecorum* Fabricius, 1794 (Diptera: Gasterophilidae) is 100% in local wild equines, accounting for more than 90% of infections by the six *Gasterophilus* spp. in the local area and suggesting that *G. pecorum* is the dominant species in KNR^1–4^. This differs from the *Gasterophilus* spp. in the rest of the world, among which *Gasterophilus intestinalis* De Geer, 1776 (Diptera: Gasterophilidae) is the dominant species^5–7^. The long-term severe infection by *G. pecorum* of Przewalski’s horse seriously hinders the progress of its wild release in KNR^3^.

*G. pecorum* oviposits on shoot tips of *Stipa caucasica* Schmalh, 1892 in KNR^8^, whereas other species of *Gasterophilus* oviposit on the hairs of equine jaw, cheeks, lips, forelegs, back, and flanks^9^ (pp. 110–128). *S. caucasica*, a high-quality forage, is a constructive plant species of the desert steppe in Xinjiang. In the local area, its phenological characteristics include germination at the beginning of March and withering yellow at the end of August^10^ (pp. 143–144). *S. caucasica* is one of the most widely distributed plants in KNR and is a favorite plant of the released Przewalski’s horses^11,12^. In contrast, captive Przewalski’s horses, which feed on alfalfa year round, are not infected by *G. pecorum*^13^*.*

Selection of the oviposition site is a critical determinant of the survival of individual offspring, which is important for successful reproduction^14^. The “mother knows best” hypothesis suggests that female phytophagous insects prefer to oviposit on hosts that improve offspring survival^15,16^. Plant volatiles are highly active, low-molecular-weight, lipophilic secondary metabolites^17,18^. Phytophagous insects locate suitable hosts on which to feed and oviposit through olfactory detection of plant volatiles so as to avoid unsuitable hosts^19,20^. *G. pecorum* often oviposits dispersively on shoot tips of *S. caucasica*^21^, and Przewalski’s horses feed on this plant more during the period when they are prone to be infected by *G. pecorum*, increasing the risk of infection (unpublished data). The proportion of male *G. pecorum* choosing *S. caucasica* is the same in different growth periods, but that of females is higher during the flourishing growth period (unpublished data).

We analyzed the volatiles composition of *S. caucasica*, *G. pecorum*’s oviposition matrix plant, during different growth periods, particularly before, during, and after *G. pecorum* oviposition. We also compared volatiles composition during the oviposition stage of *S. caucasica* and its homologous plant *Stipa orientalis* Trin., 1829, as well as another main food-source plant *Ceratoides latens* (J. F. Gmel.) Reveal et Holmgren, 1972. The findings may provide a theoretical basis for the isolation and identification of volatiles responsible for electrophysiological and olfactory responses to *G. pecorum*.

## Results

### Volatile contents of *S. caucasica* shoots during the stages of oviposition by* G. pecorum*

**Figure 1:**
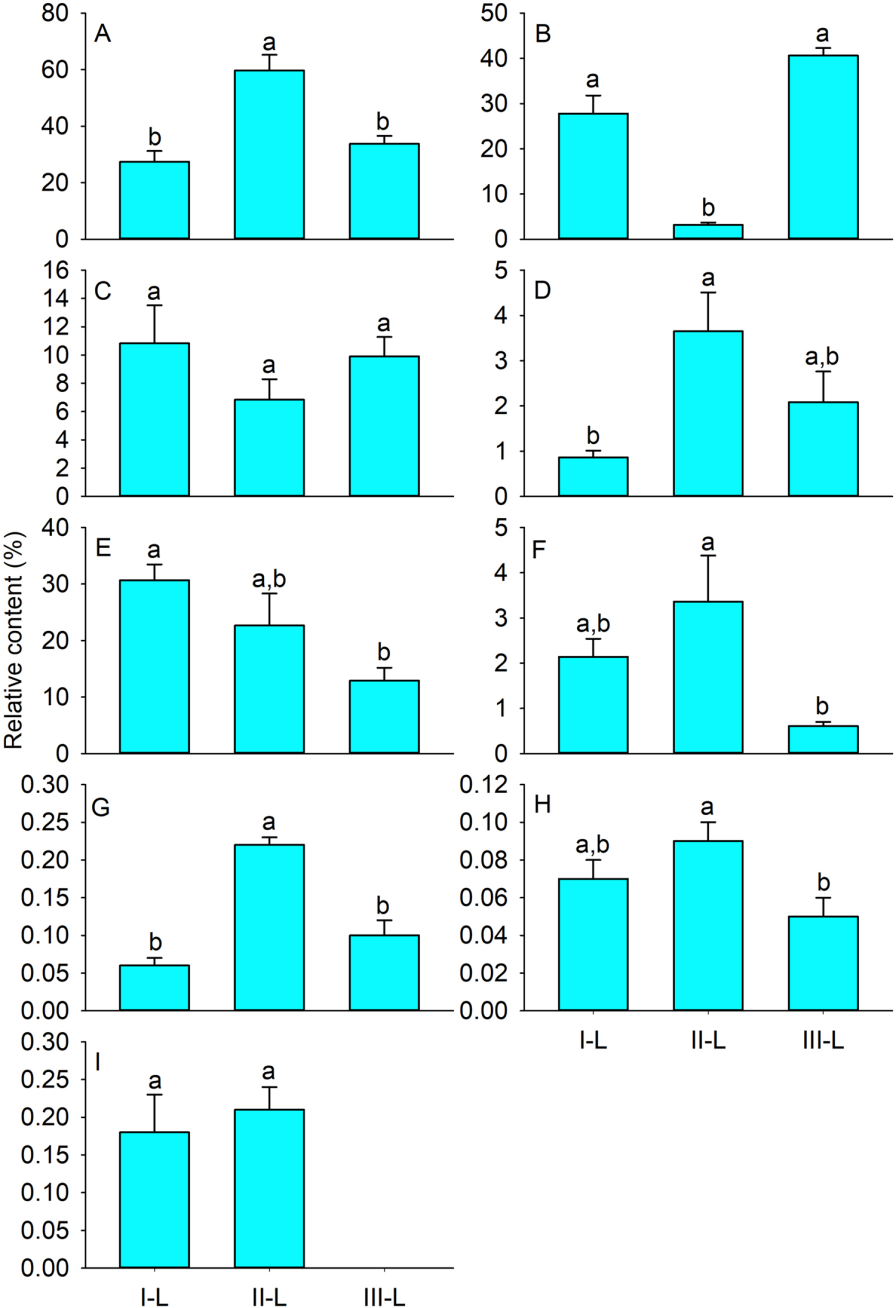
Volatiles classes from shoots of *Stipa caucasica* during preoviposition, oviposition, and postoviposition of *Gasterophilus pecorum*. I-L, II-L, and III-L represent *Stipa caucasica* shoots during the preoviposition, oviposition, and postoviposition stages of *Gasterophilus pecorum*. (**A**) alcohols, (**B**) esters, (**C**) aldehydes, (**D**) ketones, (**E**) others, (**F**) acids, (**G**) alkanes, (**H**) aromatic hydrocarbons, and (**I**) ethers. Data are mean (n = 3) ± SE. Different letters indicate significant differences at *p* < 0.05 based on the least significant difference test.

Overall, 60 volatile compounds were identified in *S. caucasica* shoots during the preoviposition (I), oviposition (II), and postoviposition (III) stages of *G. pecorum*. These comprised 16 aldehydes, 14 ketones, 12 esters, 9 alcohols, 3 alkanes, 3 aromatic hydrocarbons, 1 acid, 1 ether, and 1 other. Among them, 35 volatiles were identified in I-L, 36 in II-L, and 37 in III-L. In addition, 18 volatiles were common to I-L, II-L, and III-L; 5 to I-L and II-L; 5 to II-L and III-L; and 2 to I-L and III-L. Ten volatiles were unique to I-L, 8 to II-L, and 12 to III-L (Table 1). The main chemical classes of I-L, II-L, and III-L were alcohols, esters, and others; alcohols and others; and alcohols and esters, respectively (Fig. 1).

**Table 1:** Volatiles detected from shoots of *Stipa caucasica* during preoviposition, oviposition, and postoviposition of *Gasterophilus pecorum.*

Compounds	I-L	II-L	III-L
**Alcohols**			
1-Heptanol	0.08 ± 0.01a*	0.18 ± 0.03a	–
1-Hexanol	0.89 ± 0.05b	1.52 ± 0.06a	1.01 ± 0.09b
1-Octen-3-ol	0.17 ± 0.03	–	–
2-Hexen-1-ol,(*E*)-	0.29 ± 0.06b	0.59 ± 0.03a	0.39 ± 0.07b
2-Penten-1-ol,(*Z*)-	0.20 ± 0.05a	0.52 ± 0.11a	–
3-Hexen-1-ol,(*Z*)-	25.68 ± 3.79a	55.65 ± 5.40a	32.35 ± 2.70a
Benzenemethanol,ππ-dimethyl-	0.07 ± 0.02	–	–
1-Hexanol,2-ethyl-	–	0.73 ± 0.06	–
7-Octen-2-ol,2,6-dimethyl-	–	0.53 ± 0.12	–
**Aldehydes**			
2-Heptenal,(*Z*)-	0.10 ± 0.01a	0.19 ± 0.04a	0.17 ± 0.02a
2-Hexenal,(*E*)-	0.27 ± 0.13	–	–
2-Nonenal	0.07 ± 0.01	–	–
2-Octenal,(*E*)-	0.10 ± 0.01b	0.25 ± 0.04a	0.22 ± 0.04a,b
3-Hexenal	7.10 ± 2.97a	–	5.03 ± 0.93a
Decanal	0.78 ± 0.14b	1.20 ± 0.11a	0.65 ± 0.08b
Furfural	0.05 ± 0.01	–	–
Heptanal	0.23 ± 0.02b	0.43 ± 0.06a	0.27 ± 0.42b
Hexanal	0.62 ± 0.22a	2.38 ± 1.32a	1.16 ± 0.60a
Nonanal	1.45 ± 0.14a,b	1.90 ± 0.29a	0.96 ± 0.15b
Undecanal	0.06 ± 0.01b	0.12 ± 0.01a	0.07 ± 0.02a,b
3-Pentenal,4-methyl-	–	0.08 ± 0.01	–
2,4-Hexadienal,(*E*,*E*)-	–	0.30 ± 0.01a	0.22 ± 0.00b
Benzaldehyde	–	–	0.99 ± 0.19
2-Nonenal,(*E*)-	–	–	0.09 ± 0.02
1-Cyclohexene-1-carboxaldehyde,2,6,6-trimethyl-	–	–	0.08 ± 0.03
**Alkanes**			
Undecane,2,6-dimethyl-	0.06 ± 0.01a	0.08 ± 0.01a	0.03 ± 0.00b
Hexadecane	–	0.14 ± 0.02	–
6,7-Dioxabicyclo[3.2.1]octane,1-methyl-	–	–	0.08 ± 0.02
**Esters**			
2,2,4-Trimethyl-1,3-pentanediol diisobutyrate	0.71 ± 0.12	–	–
2(3H)-Furanone,5-ethyldihydro-	0.16 ± 0.03a	0.26 ± 0.02a	–
3-Hexen-1-ol,acetate,(*Z*)-	24.80 ± 3.74	–	–
Acetic acid hexyl ester	0.52 ± 0.05b	1.47 ± 0.30a	1.14 ± 0.07a
Acetic acid phenylmethyl ester	0.05 ± 0.01a,b	0.13 ± 0.04a	0.03 ± 0.01b
Ethyl acetate	0.46 ± 0.08a	0.39 ± 0.17a	0.07 ± 0.15a
Propanoic acid,2-methyl-,3-hydroxy-2,4,4-trimethylpentyl ester	1.12 ± 0.23	–	–
Acetic acid pentyl ester	–	0.06 ± 0.01a	0.06 ± 0.01a
3-Hexen-1-ol,formate,(*Z*)-	–	0.19 ± 0.03a	0.09 ± 0.01b
3-Cyclohexen-1-ol,acetate	–	0.66 ± 0.29a	0.37 ± 0.04a
2-Hexen-1-ol,acetate,(*E*)-	–	–	0.15 ± 0.01
3-Hexen-1-ol, acetate,(*E*)-	–	–	38.70 ± 1.65
**Ketones**			
[1,1′-Bicyclopentyl]-2-one	0.19 ± 0.04	–	–
2-Hexanone,4-methyl-	0.15 ± 0.02a	0.19 ± 0.04a	–
5-Hepten-2-one,6-methyl-	0.28 ± 0.07b	0.45 ± 0.04a,b	0.68 ± 0.18a
Acetophenone	0.16 ± 0.03a	0.17 ± 0.03a	0.07 ± 0.01b
Benzophenone	0.07 ± 0.01a	–	0.09 ± 0.03a
2(3H)-Furanone,dihydro-5-methyl-	–	0.17 ± 0.09a	0.23 ± 0.13a
2(5H)-Furanone,5-ethyl-	–	2.38 ± 0.71	–
Cyclohexanone,5-methyl-2-(1-methylethyl)-	–	0.16 ± 0.01	–
2-Undecanone,6,10-dimethyl-	–	0.14 ± 0.01	–
2-Heptanone	–	–	0.27 ± 0.07
Acetone	–	–	0.17 ± 0.09
2-Heptanone,6-methyl-	–	–	0.36 ± 0.12
2(3H)-Furanone,5-ethenyldihydro-5-methyl-	–	–	0.11 ± 0.03
2-Cyclohexen-1-one,3,5,5-trimethyl-	–	–	0.11 ± 0.05
**Aromatic hydrocarbons**			
Naphthalene,1-methyl-	0.07 ± 0.01	–	–
Naphthalene,2-methyl-	–	–	0.05 ± 0.01
1H-Indene,1-ethylidene-	–	0.09 ± 0.01	–
**Acids**			
Acetic acid	2.14 ± 0.4a,b	3.36 ± 1.02a	0.61 ± 0.09b
**Ethers**			
Ethanol,2-butoxy-	0.18 ± 0.04a	0.21 ± 0.03a	–
**Others**			
Caprolactam	30.66 ± 2.8a	22.68 ± 5.63a,b	12.90 ± 2.28b

*Data are mean (n = 3) ± SE.

Different letters indicate significant differences at *p* < 0.05 based on the least significant difference test.

Nine alcohols were identified from the three stages of oviposition on *S. caucasica*. Among them, three, *i.e.*, 3-hexen-1-ol,(*Z*)-, 1-hexanol, and 2-hexen-1-ol,(*E*)-, were common to all three stages, and two, *i.e.*, 2-penten-1-ol,(*Z*)- and 1-heptanol, were common to two stages. The relative contents of alcohols were higher in II-L (59.72%) than in III-L (33.74%; *P* = 0.009) and I-L (27.38%; *P* = 0.002), whereas III-L and I-L showed no significant difference (*P* > 0.05) (Fig. 1A). Of the alcohols, 3-hexen-1-ol,(Z)- had the highest relative contents, 25.68%, 55.65%, and 32.35% in I-L, II-L, and III-L, respectively, with no significant differences among these three (*P* > 0.05). The relative content of 1-hexanol was higher in II-L (1.52%) than in III-L (1.01%) (*P* = 0.002) or I-L (0.89%) (*P* = 0.001), whereas III-L and I-L showed no significant difference (*P* > 0.05). The relative contents of the other volatile alcohols were less than 0.8% (Table 1).

Twelve esters were identified from the three stages of *S. caucasica*. Among them, three, *i.e.*, acetic acid hexyl ester, ethyl acetate, and acetic acid phenylmethyl ester, were common to all three stages; and four, *i.e.*, 3-cyclohexen-1-ol,acetate, 2(3H)-furanone,5-ethyldihydro-, 3-hexen-1-ol,formate,(*Z*)-, and acetic acid pentyl ester, were common to two of the three stages. The relative contents of esters were lower in II-L (3.16%) than in III-L (40.61%) or I-L (27.81%) (*P* = 0.000; *P* = 0.000), whereas there was no significant difference between III-L and I-L (*P* > 0.05) (Fig. 1B). The relative contents of acetic acid hexyl ester in II-L (1.47%) and III-L (1.14%) were not significantly different (*P* > 0.05), but were higher in both than in I-L (0.52%) (*P* = 0.001 and 0.005, respectively). The relative contents of 3-hexen-1-ol,acetate,(*Z*)- (24.8%), a specific volatile of I-L, and 3-hexen-1-ol,acetate(*E*)- (38.7%), which was specific to III-L, were highest in esters in stages specifically containing them. The relative content of propanoic acid,2-methyl-,3-hydroxy-2,4,4-trimethylpentyl ester, which was detected only in I-L, was 1.12%, whereas those of the other volatiles in esters were lower than 0.8% (Table 1).

Sixteen aldehydes were identified from the three stages of *S. caucasica*. Among them, seven, *i.e.*, hexanal, nonanal, decanal, heptanal, undecanal, 2-octenal, (*E*)-, and 2-heptenal,(*Z*)-, were common to all three stages; and two, *i.e.*, 3-hexenal and 2,4-hexadienal, (*E,E*)-, were common to two of the three stages. The relative contents of aldehydes in I-L, II-L, and III-L were 10.83%, 6.84%, and 9.9%, and those of hexanal were 0.62%, 2.38%, and 1.16%, respectively; none of these differences was significant (*P* > 0.05) (Fig. 1C). The relative contents of nonanal in I-L (1.45%) and II-L (1.9%) did not differ significantly (*P* > 0.05), and both were higher than that in III-L (0.96%) (*P* > 0.05 and* P* = 0.018, respectively). The relative content of decanal was higher in II-L (1.20%) than in I-L (0.78%) (*P* = 0.043) or III-L (0.65%) (*P* = 0.016), but those in I-L and III-L did not differ significantly (*P* > 0.05). The following two volatiles were present in two of the three stages: 3-hexenal, with higher content in I-L (7.10%) than in III-L (5.03%) (*P* > 0.05); and 2,4-hexadienal,(*E*,*E*)-, with content higher in II-L (0.3%) than in III-L (0.22%) (*P* = 0.00). Benzaldehyde was specific to III-L (0.99%), with the relative contents of other volatile aldehydes < 0.5% (Table 1).

Fourteen ketones were identified from the three stages of *S. caucasica*. Among them, two, *i.e.*, 5-hepten-2-one,6-methyl- and acetophenone, were common to all three stages; and three, *i.e.*, 2(3H)-furanone,dihydro-5-methyl-, 2-hexanone,4-methyl-, and benzophenone, were common to two of the three stages. The relative contents of ketones were lower in I-L (0.86%) than in II-L (3.65%) or III-L (2.08%) (*P* = 0.022 and *P* > 0.05), with no significant difference between II-L and III-L (*P* > 0.05) (Fig. 1D). The content of 2(5H)-Furanone,5-ethyl- was specific to II-L (2.38%), and the relative contents of the other ketones were < 0.7% (Table 1).

The relative content of caprolactam, the only volatile in the class ‘others,’ was 30.66% and 22.68% in I-L and II-L, respectively; there was no significant difference between values for I-L and II-L (*P* > 0.05), and both were higher than those for III-L (12.9%) (*P* = 0.017 and *P* > 0.05, respectively) (Fig. 1E). The relative content of acetic acid, the only volatile in the class of acids, was lower in III-L (0.61%) than in II-L (3.36%) or I-L (2.14%) (*P* = 0.022 and *P* > 0.05, respectively); there was no significant difference between the latter two (*P* > 0.05). The relative contents of alkanes, aromatic hydrocarbons, and ethers were less than 0.22% (Fig. 1G–I). These included three alkanes, one in I-L and two each in II-L and III-L; three aromatic hydrocarbons, one of them specific to each stage; and one ether, which was not found in III-L (Table 1).

The five volatile compounds with the highest relative contents, in order, during the three stages of *S. caucasica* were as follows: I-L, caprolactam (30.66%) > 3-hexen-1-ol,(*Z*)- (25.68%) > 3-hexen-1-ol,acetate,(*Z*)- (24.8%) > 3-hexenal (7.1%) > acetic acid (2.14%); II-L, 3-hexen-1-ol,(*Z*)- (55.65%) > caprolactam (22.68%) > acetic acid (3.36%) > hexanal (2.38%) = 2(5H)-furanone,5-ethyl- (2.38%); III-L, 3-hexen-1-ol,acetate,(*E*)- (38.7%) > 3-hexen-1-ol,(*Z*)- (32.35%) > caprolactam (12.9%) > 3-hexenal (5.03%) > hexanal (1.16%) (Table 1). A total of eight volatiles were detected: two (*i.e.*, 3-hexen-1-ol,(*Z*)- and caprolactam) were common to the three stages, and three (*i.e.*, acetic acid, 3-hexenal, and hexanal) to two of the three stages. Finally, 2(5H)-furanone,5-ethyl- was in the top 5 volatile compounds of only II-L.

### Relative contents of volatiles in three plant species during the oviposition stage of *G. pecorum*

**Figure 2:**
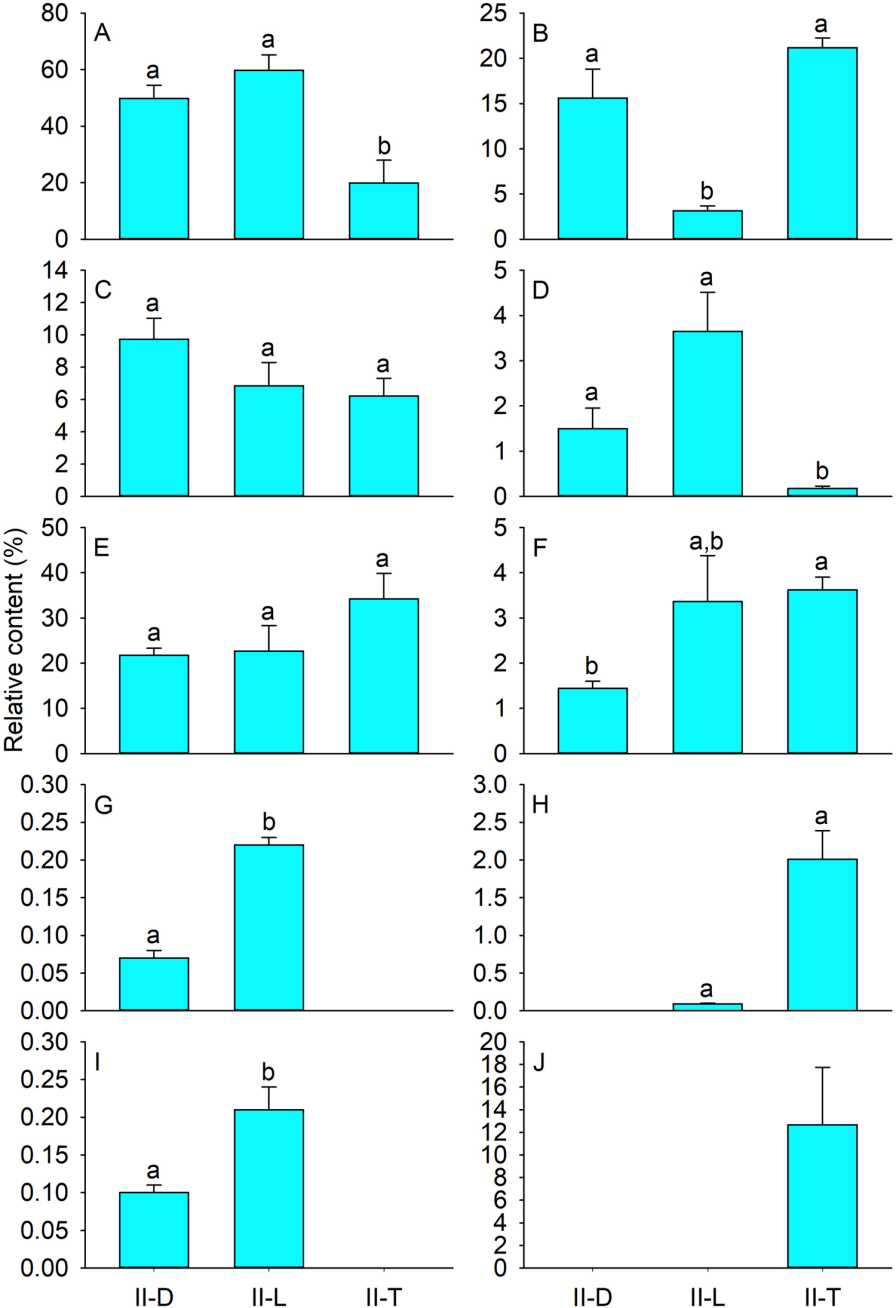
Volatiles classes from shoots of three plant species during oviposition of *Gasterophilus pecorum.* II-D, II-L, and II-T represent shoots of *Stipa orientalis*, *Stipa caucasica*, and *Ceratoides latens* during the oviposition stage of *Gasterophilus pecorum*. (**A**) alcohols, (**B**) esters, (**C**) aldehydes, (**D**) ketones, (**E**) others, (**F**) acids, (**G**) alkanes, (**H**) aromatic hydrocarbons, (**I**) ethers, and (**J**) alkenes. Data are mean (n = 3) ± SE. Different letters indicate significant differences at *p* < 0.05 based on the least significant difference test.

During the oviposition stage of *G. pecorum*, a total of 60 volatiles were identified in *S. orientalis* (II-D), *S. caucasica* (II-L), and *C. latens* (II-T). These comprised 18 esters, 13 aldehydes, 11 alcohols, 10 ketones, 2 alkanes, 2 aromatic hydrocarbons, 1 acid, 1 alkene, 1 ether, and 1 other. Of these, 35 were identified in II-D, 36 in II-L, and 27 in II-T. In addition, 11 were common to II-D, II-L, and II-T, 14 to II-D and II-L, and 2 to II-L and II-T; 10 were unique to II-D, 9 to II-L, and 14 to II-T (Table 2). The main chemical classes of II-D and II-L were alcohols and others, and those of II-T were alcohols, esters, and others (Fig. 2).

**Table 2:** Volatiles detected from shoots of three plant species during oviposition of *Gasterophilus pecorum.*

Compound	II-D	II-L	II-T
**Alcohols**			
1-Heptanol	0.14 ± 0.03a*	0.18 ± 0.03a	–
1-Hexanol	1.67 ± 0.35a	1.52 ± 0.06a	2.79 ± 0.85a
1-Octen-3-ol	0.25 ± 0.14	–	–
2-Hexen-1-ol,(*E*)-	0.72 ± 0.18a	0.59 ± 0.03a	2.66 ± 1.12a
2-Penten-1-ol,(*Z*)-	0.23 ± 0.05a	0.52 ± 0.11a	–
3-Hexen-1-ol,(*Z*)-	44.64 ± 4.51a	55.65 ± 5.40a	14.28 ± 6.32b
1-Hexanol,2-ethyl-	0.45 ± 0.09a	0.73 ± 0.06a	–
7-Octen-2-ol,2,6-dimethyl-	–	0.53 ± 0.12	–
Phenylethyl alcohol	0.11 ± 0.05	–	–
3-Hexen-1-ol	1.57 ± 0.51	–	–
1-Hexanol,3-methyl-	–	–	0.20 ± 0.05
**Aldehydes**			
2-Heptenal,(*Z*)-	0.09 ± 0.03a	0.19 ± 0.04a	–
2-Octenal,(*E*)-	0.13 ± 0.04a	0.25 ± 0.04a	–
3-Hexenal	6.57 ± 0.11	–	–
Decanal	0.69 ± 0.06a	1.20 ± 0.11a	1.04 ± 0.37a
Heptanal	0.25 ± 0.06a	0.43 ± 0.06a	0.38 ± 0.10a
Hexanal	0.82 ± 0.82a	2.38 ± 1.32a	1.90 ± 0.10a
Nonanal	0.94 ± 0.16a	1.90 ± 0.29a	1.80 ± 0.56a
Undecanal	0.05 ± 0.02a	0.12 ± 0.01a	–
3-Pentenal,4-methyl-	–	0.08 ± 0.01	—
2,4-Hexadienal,(*E*,*E*)-	0.15 ± 0.02a	0.30 ± 0.01a	–
Benzaldehyde	–	–	0.94 ± 0.25
1-Cyclohexene-1-carboxaldehyde,2,6,6-trimethyl-	0.06 ± 0.02	–	–
2-Hexenal	–	–	0.15 ± 0.05
**Alkanes**			
Undecane,2,6-dimethyl-	0.07 ± 0.01a	0.08 ± 0.01a	–
Hexadecane	–	0.14 ± 0.02	–
**Esters**			
2(3H)-Furanone,5-ethyldihydro-	0.27 ± 0.03b	0.26 ± 0.02b	0.71 ± 0.07a
3-Hexen-1-ol,acetate,(*Z*)-	13.13 ± 2.87	–	–
Acetic acid hexyl ester	0.4 ± 0.06a	1.47 ± 0.30a	4.25 ± 1.67a
Acetic acid phenylmethyl ester	–	0.13 ± 0.04	–
Ethyl acetate	–	0.39 ± 0.17a	0.63 ± 0.27a
Propanoic acid,2-methyl-,3-hydroxy-2,4,4-trimethylpentyl ester	1.07 ± 0.41	–	–
Acetic acid pentyl ester	–	0.06 ± 0.01a	0.14 ± 0.04a
3-Hexen-1-ol,formate,(*Z*)-	–	0.19 ± 0.03a	–
3-Cyclohexen-1-ol,acetate	–	0.66 ± 0.29a	–
Propanoic acid,2-methyl-,2,2-dimethyl-1-(2-hydroxy-1-methylethyl) propyl ester	0.75 ± 0.34	–	–
1-Butanol,3-methyl-,acetate	–	–	0.70 ± 0.12
2-Penten-1-ol,acetate,(*Z*)-	–	–	0.61 ± 0.19
2-Hexenoic acid,methyl ester	–	–	0.60 ± 0.34
Benzoic acid,methyl ester	–	–	1.88 ± 0.47
Methyl salicylate	–	–	2.52 ± 0.39
*cis*-3-Hexenyl isovalerate	–	–	8.45 ± 1.48
Butanoic acid,3-methyl-,hexyl ester	–	–	0.39 ± 0.03
*trans*-2-Hexenyl valerate	–	–	0.31 ± 0.03
**Ketones**			
2-Hexanone,4-methyl-	0.21 ± 0.09a	0.19 ± 0.04a	–
5-Hepten-2-one,6-methyl-	0.39 ± 0.10a	0.45 ± 0.04a	–
Acetophenone	0.07 ± 0.02a	0.17 ± 0.03b	–
2(3H)-Furanone,dihydro-5-methyl-	0.35 ± 0.13a	0.17 ± 0.09a	–
2(5H)-Furanone,5-ethyl-	–	2.38 ± 0.71	–
Cyclohexanone,5-methyl-2-(1-methylethyl)-	–	0.16 ± 0.01	–
2-Undecanone,6,10-dimethyl-	0.16 ± 0.07a	0.14 ± 0.01a	–
2-Heptanone,6-methyl-	0.19 ± 0.05	–	–
2(3H)-Furanone,5-ethenyldihydro-5-methyl-	0.13 ± 0.03	–	–
2-Pentadecanone,6,10,14-trimethyl-	–	–	0.18 ± 0.05
**Aromatic hydrocarbons**			
1H-Indene,1-ethylidene-	–	0.09 ± 0.01	–
Benzene,1-methyl-2-(1-methylethyl)-	–	–	2.01 ± 0.38
**Acids**			
Acetic acid	1.44 ± 0.16b	3.36 ± 1.02a,b	3.62 ± 0.28a
**Alkenes**			
1,3,6-Octatriene,3,7-dimethyl-	–	–	12.67 ± 5.08
**Ethers**			
Ethanol,2-butoxy-	0.10 ± 0.01a	0.21 ± 0.03b	–
**Others**			
Caprolactam	21.76 ± 1.57a	22.68 ± 5.63a	34.20 ± 5.68a

*Data are mean (n = 3) ± SE.

Different letters indicate significant differences at *p* < 0.05 based on the least significant difference test.

Eleven alcohols were identified from the three plant species during the oviposition stage of *G. pecorum*. Among them, three, *i.e.*, 3-hexen-1-ol,(*Z*)-, 1-hexanol, and 2-hexen-1-ol,(*E*)-, were common to all three species, and three, *i.e.*, 1-hexanol,2-ethyl-, 2-penten-1-ol,(*Z*)-, and 1-heptanol, were common to two of the three. The relative contents of alcohols were lower in II-T (19.93%) than in II-L (59.72%) or II-D (49.79%) (*P* = 0.004 and 0.015, respectively), with no significant difference II-L and II-D (*P* > 0.05) (Fig. 2A). The relative content of 3-hexen-1-ol,(Z)- was lower in II-T (14.28%) than in II-L (55.65%) or II-D (44.64%) (*P* = 0.002 and 0.008), but there was not significant difference between II-L and II-D (*P* > 0.05). The relative contents of 1-hexanol and 2-hexen-1-ol,(*E*)- in II-D, II-L, and II-T were 1.67%, 1.52%, 2.79%, and 0.72%, and 0.59% and 2.66%, respectively; these differences were not significant (*P* > 0.05). Finally, 3-hexen-1-ol was specific to II-D (1.57%), and the relative contents of other alcohols were < 0.8% (Table 2).

Eighteen esters were identified from three plants. Among them, two, *i.e.*, acetic acid hexyl ester and 2(3H)-furanone,5-ethyldihydro- were common to all three plants, and two, *i.e.*, ethyl acetate and acetic acid pentyl ester, were common to two. The relative contents of esters were lower in II-L (3.16%) than in II-T (21.19%) (*P* = 0.000) or II-D (15.61%) (*P* = 0.001), with no significant difference between II-D and II-T (*P* > 0.05) (Fig. 2B). The relative content of acetic acid hexyl ester in II-D, II-L, and II-T was 0.4%, 1.47%, and 4.25%, respectively; these differences were not significant (*P* > 0.05). The relative content of 2(3H)-furanone, 5-ethyldihydro- was higher in II-T (0.71%) than in II-D (0.27%) or II-L (0.26%) (*P* = 0.000; *P* = 0.000), but II-D and II-L were not significantly different (*P* > 0.05). Both 3-hexen-1-ol,acetate,(Z)- (13.13%) and propanoic acid,2-methyl-,3-hydroxy-2,4,4-trimethylpentyl ester (1.07%) were unique to II-D, and benzoic acid methyl ester (1.88%), methyl salicylate (2.52%), and *cis*-3-hexenyl isovalerate (8.45%) were all unique to II-T. The relative contents of other esters were < 0.8% (Table 2).

Thirteen aldehydes were identified from the three plants. Among them, four, *i.e.*, hexanal, nonanal, decanal, and heptanal, were common to all three plants, and four, *i.e.*, 2-octenal,(*E*)-, 2-heptenal,(Z)-, 2,4-hexadienal,(*E,E*)- and undecanal, were common to two of the three species. The relative contents of aldehydes in II-D, II-L, and II-T were 9.74%, 6.84%, and 6.21%, respectively; these differences were not significant (*P* > 0.05) (Fig. 2C). The relative contents of hexanal, nonanal, decanal, and heptanal were 0.25–2.38% and were higher in II-L than in II-D or II-T, although the differences were not significant (*P* > 0.05). Finally, 3-hexenal (6.57%) was unique to II-D, and benzaldehyde (0.94%) to II-T. The relative contents of other aldehydes were < 0.5% (Table 2).

The relative contents of ketones were lower in II-T (0.18%) than in II-L (3.65%) or II-D (1.5%) (*P* = 0.000 and 0.001), but the difference between II-L and II-D was not significant (*P* > 0.05) (Fig. 2D). Five ketones, *i.e.*, 5-hepten-2-one,6-methyl-, 2(3H)-furanone,dihydro-5-methyl-, 2-hexanone,4-methyl-, 2-undecanone,6,10-dimethyl-, and acetophenone, were common to II-D and II-L, and 2(5H)-furanone,5-ethyl- (2.38%) was unique to II-L. The relative contents of other ketones were < 0.5% (Table 2).

The only volatile in the class ‘others’ was caprolactam, and its relative content in II-D, II-L, and II-T was 21.76%, 22.68%, and 34.2%, respectively, with no significant differences (*P* > 0.05) (Fig. 2E). Acetic acid was the only substance in the class ‘acids,’ and its relative content was lower in II-D (1.44%) than in II-T (3.62%) (*P* = 0.046) or II-L (3.36%) (*P* > 0.05); contents in II-T and II-L did not differ significantly (*P* > 0.05). The only alkene, 1,3,6-Octatriene,3,7-dimethyl-, was unique to II-T (12.67%). The relative contents of other alkanes and ethers were < 0.3% (Fig. 2G, I); these included two alkanes and one ether, none of which was identified in II-T. Two aromatic hydrocarbons were identified, one of which, benzene,1-methyl-2-(1-methylethyl)- (2.01%), was unique to II-T (Table 2).

The top five volatile compounds from the three plant species during the oviposition stage of *G. pecorum* were, in order: II-D, 3-hexen-1-ol,(*Z*)- (44.64%) > caprolactam (21.76%) > 3-hexen-1-ol,acetate,(*Z*)- (13.13%) > 3-hexenal (6.57%) > 1-hexanol (1.67%); II-L, 3-hexen-1-ol,(*Z*)- (55.65%) > caprolactam (22.68%) > acetic acid (3.36%) > hexanal (2.38%) = 2(5H)-furanone,5-ethyl- (2.38%); II-T, caprolactam (34.2%) > 3-hexen-1-ol,(*Z*)- (14.28%) > 1,3,6-octatriene,3,7-dimethyl- (12.67%) > *cis*-3-hexenyl isovalerate (8.45%) > acetic acid hexyl ester (4.25%) (Table 2). Eleven volatiles were included: two (3-hexen-1-ol,(*Z*)- and caprolactam) were common to all three plant species; the other nine were in the top five of only one species.

### Relative contents of volatiles from *S. caucasica* in different growth periods

**Figure 3:**
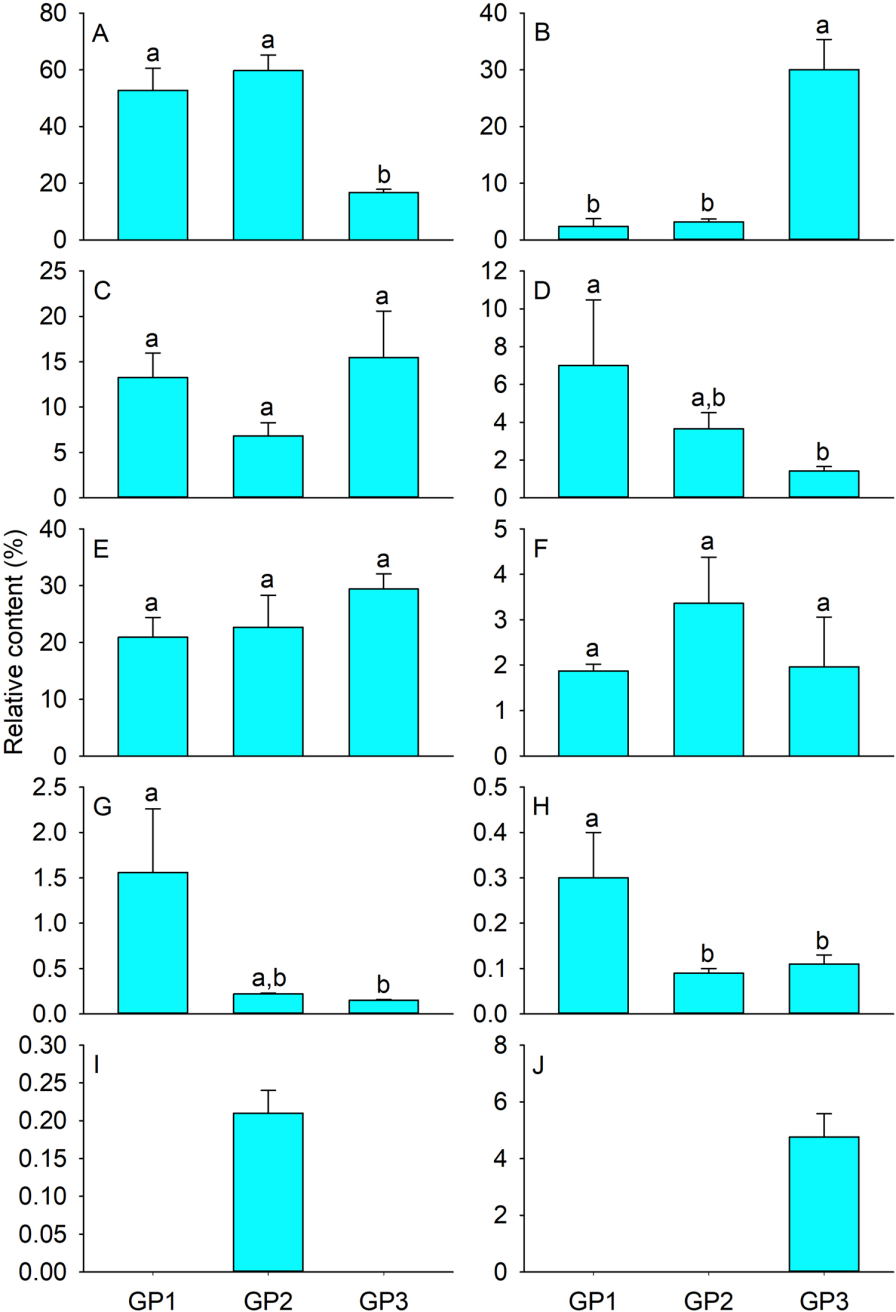
Volatiles classes from shoots of *Stipa caucasica* during its different growth periods. GP1, GP2, and GP3 represent *Stipa caucasica* shoots during the early, flourishing, and late growth periods, respectively. Note that GP2 was actually the same sample as II-L in Figs. 1 and 2. Thus, the three groups had a total of seven rather than nine samples. (**A**) alcohols, (**B**) esters, (**C**) aldehydes, (**D**) ketones, (**E**) others, (**F**) acids, (**G**) alkanes, (**H**) aromatic hydrocarbons, (**I**) ethers, and (**J**) alkenes. Data are mean (n = 3) ± SE. Different letters indicate significant differences at *p* < 0.05 based on the least significant difference test.

From *S. caucasica* at the early, flourishing, and late growth periods (GP1, GP2, and GP3, respectively), a total of 69 volatile compounds were identified. These comprised 17 ketones, 13 aldehydes, 11 esters, 10 alcohols, 4 alkanes, 4 aromatic hydrocarbons, 2 acids, 2 alkenes, 1 ether, and 5 others. Of these, 35 were found in GP1, 36 in GP2, and 40 in GP3. In addition, 11 were common to all three stages, 10 to both GP2 and GP3, 6 to both GP1 and GP2, and 4 to both GP1 and GP3; 14 were unique to GP1, 9 to GP2, and 15 to GP3 (Table 3). The main chemical classes of GP1 and GP2 were alcohols and others, and those of GP3 were esters and others (Fig. 3).

**Table 3:** Volatiles detected from shoots of *Stipa caucasica* during its different growth periods.

Compounds	GP1	GP2	GP3
**Alcohols**			
1-Heptanol	–	0.18 ± 0.03a	0.07 ± 0.01b
1-Hexanol	1.98 ± 0.27a*	1.52 ± 0.06a	0.59 ± 0.03b
2-Hexen-1-ol,(*E*)-	0.66 ± 0.10a	0.59 ± 0.03a	–
2-Penten-1-ol,(*Z*)-	–	0.52 ± 0.11a	0.16 ± 0.04b
3-Hexen-1-ol,(*Z*)-	49.50 ± 7.87a	55.65 ± 5.40a	15.42 ± 1.15b
Benzenemethanol,ππ-dimethyl-	–	–	0.14 ± 0.02
1-Hexanol,2-ethyl-	–	0.73 ± 0.06	–
7-Octen-2-ol,2,6-dimethyl-	–	0.53 ± 0.12a	0.09 ± 0.05b
1-Butanol,2-methyl-	0.39 ± 0.01	–	–
Phenylethyl alcohol	0.22 ± 0.03a	–	0.23 ± 0.01a
**Aldehydes**			
2-Heptenal,(*Z*)-	–	0.19 ± 0.04a	0.18 ± 0.04a
2-Hexenal,(*E*)-	–	–	0.39 ± 0.13
2-Octenal,(*E*)-	–	0.25 ± 0.04a	0.15 ± 0.03a
3-Hexenal	5.50 ± 3.78a	–	6.13 ± 1.49a
Decanal	1.38 ± 0.12a	1.20 ± 0.11a	1.06 ± 0.06a
Heptanal	0.86 ± 0.01a	0.43 ± 0.06b	–
Hexanal	4.77 ± 1.34a	2.38 ± 1.32a	1.51 ± 0.76a
Nonanal	–	1.90 ± 0.29a	1.43 ± 0.12a
Undecanal	0.20 ± 0.01a	0.12 ± 0.01b	0.10 ± 0.01b
3-Pentenal,4-methyl-	–	0.08 ± 0.01	–
2,4-Hexadienal,(*E*,*E*)-	0.40 ± 0.05a	0.30 ± 0.01a	–
Dodecanal	0.14 ± 0.04	–	–
2-Hexenal	–	–	4.51 ± 0.51
**Alkanes**			
Undecane,2,6-dimethyl-	0.67 ± 0.40a	0.08 ± 0.01b	0.06 ± 0.01b
Hexadecane	–	0.14 ± 0.02	–
Tridecane	0.89 ± 0.30	–	–
Nonane,2,2,4,4,6,8,8-heptamethyl-	–	–	0.09 ± 0.01
**Esters**			
2(3H)-Furanone,5-ethyldihydro-	–	0.26 ± 0.02a	0.10 ± 0.00b
3-Hexen-1-ol,acetate,(*Z*)-	–	–	28.42 ± 5.08
Acetic acid hexyl ester	0.52 ± 0.09b	1.47 ± 0.30a	0.98 ± 0.22a,b
Acetic acid phenylmethyl ester	–	0.13 ± 0.04a	0.12 ± 0.04a
Ethyl acetate	0.28 ± 0.08a	0.39 ± 0.17a	–
Propanoic acid,2-methyl-,3-hydroxy-2,4,4-trimethylpentyl ester	1.48 ± 1.39	–	–
Acetic acid pentyl ester	–	0.06 ± 0.01a	0.11 ± 0.02a
3-Hexen-1-ol,formate,(*Z*)-	0.09 ± 0.02b	0.19 ± 0.03a	0.08 ± 0.04b
3-Cyclohexen-1-ol,acetate	–	0.66 ± 0.29	–
3-Hexenoic acid,methyl ester,(*E*)-	–	–	0.13 ± 0.05
Acetic acid,2-ethylhexyl ester	–	–	0.08 ± 0.02
**Ketones**			
2-Hexanone,4-methyl-	–	0.19 ± 0.04	–
5-Hepten-2-one,6-methyl-	0.70 ± 0.13a	0.45 ± 0.04a,b	0.33 ± 0.05b
Acetophenone	–	0.17 ± 0.03a	0.14 ± 0.02a
Benzophenone	0.16 ± 0.02	–	–
2(3H)-Furanone,dihydro-5-methyl-	0.26 ± 0.03a	0.17 ± 0.09a	–
2(5H)-Furanone,5-ethyl-	–	2.38 ± 0.71	–
Cyclohexanone,5-methyl-2-(1-methylethyl)-	–	0.16 ± 0.01	–
2-Undecanone,6,10-dimethyl-	3.12 ± 0.82a	0.14 ± 0.01b	–
Acetone	0.18 ± 0.02a	–	0.31 ± 0.11a
2-Heptanone,6-methyl-	0.70 ± 0.05	–	–
2(3H)-Furanone,5-ethenyldihydro-5-methyl-	0.82 ± 0.69	–	–
2,4-Pentanedione	0.24 ± 0.03	–	–
Bicyclo[2.2.1]heptan-2-one,1,7,7-trimethyl-,(1R)-	0.74 ± 0.22	–	–
2-Pentadecanone,6,10,14-trimethyl-	0.08 ± 0.01	–	–
2-Butanone,3-hydroxy-	–	–	0.22 ± 0.03
Bicyclo[2.2.2]oct-5-en-2-one	–	–	0.30 ± 0.07
Bicyclo[2.2.1]heptan-2-one,1,7,7-trimethyl-,(1S)-	–	–	0.13 ± 0.03
**Aromatic hydrocarbons**			
1H-Indene,1-ethylidene-	–	0.09 ± 0.01	–
Naphthalene,2-methyl-	0.09 ± 0.03	–	–
Benzene,1,2,4-trimethyl-	0.22 ± 0.07	–	–
Benzene,1-ethenyl-4-methoxy-	–	–	0.11 ± 0.02
**Acids**			
Acetic acid	1.87 ± 0.15a,b	3.36 ± 1.02a	0.97 ± 0.33b
Propanoic acid,2-methyl-,2,2-dimethyl-1-	–	–	1.00 ± 0.89
**Alkenes**			
1,3,6-Octatriene,3,7-dimethyl-	–	–	4.70 ± 0.80
2,6-Dimethyl-1,3,5,7-octatetraene,(*E*,*E*-)	–	–	0.07 ± 0.01
**Ethers**			
Ethanol,2-butoxy-	–	0.21 ± 0.03	–
**Others**			
Caprolactam	19.78 ± 3.48a	22.68 ± 5.63a	28.80 ± 2.78a
Furan,2,3-dihydro-5-methyl-	0.12 ± 0.02	–	–
Furan,2-ethyl-	0.40 ± 0.06	–	–
Furan,2-pentyl-	0.59 ± 0.10a	–	0.43 ± 0.07a
Butanenitrile,2-methyl-	–	–	0.18 ± 0.05

*Data are mean (n = 3) ± SE.

Different letters indicate significant differences at *p* < 0.05 based on the least significant difference test.

Ten alcohols were identified. Among them, two, *i.e.*, 3-hexen-1-ol,(*Z*)- and 1-hexanol, were common to all three periods, and five, *i.e.*, 2-hexen-1-ol,(*E*)-, 7-octen-2-ol,2,6-dimethyl-, 2-penten-1-ol,(*Z*)-, phenylethyl alcohol, and 1-heptanol, were common to two of the three periods. The relative contents of alcohols were lower in GP3 (16.7%) than in GP1 (52.75%) or GP2 (59.72%) (*P* = 0.004 and 0.002, respectively) (Fig. 3A); the latter two values were not significantly different (*P* > 0.05). The 3-hexen-1-ol,(Z)- content, which was the highest among all alcohols, was lower in GP3 (15.42%) than in GP1 (49.5%) or GP2 (55.65%) (*P* = 0.005 and 0.002, respectively); the latter two did not differ significantly (*P* > 0.05). The relative content of 1-hexanol was lower in GP3 (0.59%) than in GP1 (1.98%) or GP2 (1.52%) (*P* = 0.001 and 0.007, respectively); the latter two were not significantly different (*P* > 0.05). The relative contents of other alcohols were < 0.8% (Table 3).

Eleven esters were identified. Among them, two, *i.e.*, acetic acid hexyl ester and 3-hexen-1-ol,formate,(*Z*)-, were common to all three periods, and four, *i.e.*, ehthyl acetate, 2(3H)-furanone,5-ethyldihydro-, acetic acid phenylmethyl ester, and acetic acid pentyl ester, were common to two of the three. The relative contents of esters were higher in GP3 (30.02%) than in GP1 (2.37%) or GP2 (3.16%) (*P* = 0.000; *P* = 0.000), but GP1 and GP2 did not differ significantly (*P* > 0.05) (Fig. 3B). The relative content of acetic acid hexyl ester was higher in GP2 (1.47%) than in GP1 (0.52%) (*P* = 0.022) or GP3 (0.98%) (*P* > 0.05), with no significant difference between GP1 and GP3 (*P* > 0.05). Propanoic acid,2-methyl-,3-hydroxy-2,4,4-trimethylpentyl ester (1.48%) was unique to GP1, and 3-hexen-1-ol,acetate,(*Z*)- (28.42%) to GP3. The relative contents of other esters were < 0.7% (Table 3).

Five ‘others’ were identified, of which caprolactam was common to all three growth periods. The relative contents of the ‘others’ were 20.9%, 22.68%, and 29.41%, and those of caprolactam were 19.78%, 22.68%, and 28.80% in GP1, GP2, and GP3, respectively; these differences were not significant (*P* > 0.05) (Fig. 3E). The relative contents of the remaining four ‘others’ were < 0.6% (Table 3).

Thirteen aldehydes were identified. Among them, three, *i.e.*, hexanal, decanal, and undecanal, were common to all three periods, and six; *i.e.*, 3-hexenal, nonanal, heptanal, 2-octenal,(*E*)-, 2,4-hexadienal,(*E*,*E*)-, and 2-heptenal,(*Z*)-, were common to two of the three periods. The relative contents of aldehydes in GP1, GP2, and GP3 were not significantly different, at 13.25%, 6.84%, and 15.46%, respectively (*P* > 0.05) (Fig. 3C). The relative contents of hexanal and decanal decreased with growth period from 4.77% and 1.38% to 1.51% and 1.06%, respectively; but there were no significant differences between periods (*P* > 0.05). Six volatiles were common to two of the three periods. There were no significant differences between the relative contents of 3-hexenal in GP3 (6.13%) and in GP1 (5.50%) (*P* > 0.05) or between those of nonanal in GP2 (1.90%) and GP3 (1.43%) (*P* > 0.05). Finally, 2-hexenal (4.51%) was unique to GP3, and the relative contents of other aldehydes were < 0.9% (Table 3).

Seventeen ketones were identified, of which 5-hepten-2-one,6-methyl- was common to all three periods, and four; *i.e.*, 2-undecanone,6,10-dimethyl-, acetone, 2(3H)-furanone,dihydro-5-methyl-, and acetophenone, were common to two of the three periods. The relative contents of ketones were higher in GP1 (7%) than GP3 (1.42%) (*P* = 0.020), but there was no significant difference between the content in GP2 (3.65%) and in GP1 or GP3 (both *P* > 0.05) (Fig. 3D). The relative content of 5-hepten-2-one, 6-methyl- was higher in GP1 (0.7%) than in GP3 (0.33%) (*P* = 0.020), with no significant difference between that in GP2 (0.45%) and that in GP1 or GP3 (both *P* > 0.05). The relative content of 2-undecanone,6,10-dimethyl- was higher in GP1 (3.12%) than in GP2 (0.14%) (*P* = 0.05). Finally, 2(5H)-furanone,5-ethyl- (2.38%) was specific to GP2, and the relative contents of other ketones were < 0.9% (Table 3).

Two acids were identified, and their relative contents in GP1, GP2, and GP3 were 1.87%, 3.36%, and 1.96%, respectively; none of these differences was significant (*P* > 0.05) (Fig. 3F). The relative content of acetic acid, which was common to all three periods, was higher in GP2 (3.36%) than in GP3 (0.97%) (*P* = 0.035), but there was no significant difference between GP1 (1.87%) and GP2 or GP3 (both *P* > 0.05). The other acid, propanoic acid,2-methyl-,2,2-dimethyl-1- (1%), was specific to GP3 (Table 3).

Four alkanes were identified, and the relative contents of individual alkanes ranged from 0.06% to 0.89%. The relative contents of all alkanes were higher in GP1 (1.56%) than in GP3 (0.15%) (*P* = 0.022), with no significant difference between GP2 (0.22%) and GP1 or GP3 (both *P* > 0.05) (Fig. 3G). Two alkenes were found only in GP3; they had a total relative content of 4.76% (Fig. 3J); one, 1,3,6-octatrine,3,7-dimethyl-, accounted for 4.70% of this total. The relative aromatic hydrocarbon and ether contents were < 0.4% (Fig. 3H and I). Four of the former were detected, each unique to one of the three periods; one of the latter was identified, only in GP2 (Table 3).

The top five volatiles from the three growth periods of *S. caucasica* were as follows: GP1, 3-hexen-1-ol,(*Z*)- (49.5%) > caprolactam (19.78%) > 3-hexenal (5.5%) > hexanal (4.77%) > 2-undecanone,6,10-dimethyl- (3.12%); GP2, 3-hexen-1-ol,(*Z*)- (55.65%) > caprolactam (22.68%) > acetic acid (3.36%) > hexanal (2.38%) = 2(5H)-furanone,5-ethyl-(2.38%); GP3, caprolactam (28.8%) > 3-hexen-1-ol,acetate,(*Z*)- (28.42%) > 3-hexen-1-ol,(*Z*)- (15.42%) > 3-hexenal (6.13%) > 1,3,6-octatriene,3,7-dimethyl- (4.70%) (Table 3). Overall, nine volatiles were detected: two (3-hexen-1-ol,(*Z*)- and caprolactam) were in the top five in all three growth periods, two (3-hexenal and hexanal) in two growth periods, and the other five were in the top five of in only one of the three growth periods.

## Discussion

*G*. *pecorum* is one of the most pathogenic species of *Gasterophilus* spp., which is distributed in Europe, Africa, and Asia. A large number of *G. pecorum* larvae attached to a horse can cause inflammation and dysphagia and may result in death due to esophageal contraction^22^ (pp. 526–528). A previous study^9^ showed that *G. pecorum* was the only *Gasterophilus* spp. that oviposited on grass. Liu et al.^8^ found that *G. pecorum* oviposited on tips of *S. caucasica* shoots in KNR, whereas our team also found that *S. orientalis* of the same genus was rare in the area, and eggs of *G. pecorum* were not found on its tip*.* Selection of an oviposition site by *G. pecorum* might be related to the behavior of Przewalski’s horses as well as the availability of food and water^8^. *S. caucasica* is the dominant plant species in KNR, and *S. caucasica* and *C. latens* are the main food sources for Przewalski’s horse^12^. During infection by *G. pecorum*, the proportion of Przewalski’s horse and Mongolian wild ass feeding on *S. caucasica* is increasing (unpublished data). High densities of equine feces and *G. pecorum* eggs are found in the vicinity of water sources, making these areas the main transmission sites of *Gasterophilus* myiasis in the local area^8,23^. The Przewalski’s horse drinks water daily, and its activity range is restricted to the vicinity of water sources^24,25^. In contrast, Mongolian wild ass is more drought tolerant and has a wider activity range^26^, which may explain why a greater number of Przewalski’s horses than of Mongolian wild asses is infected by *Gasterophilus* spp.

The lifespan of adult *G. pecorum* is 1–4 days; its longest survival time is shorter than that of other species of the genus. Each *G. pecorum* lays 1,300–2,425 eggs, more than other species of the genus^9^. Chereshnev^27^ (pp. 765–768) reported that in Kazakhstan, *G. pecorum* lays 10–15 eggs at each site of grass, whereas *G. pecorum* lays 1–10 eggs at each *S. caucasica* tip in the wild release area of the Przewalski’s horse in Xinjiang, with an average of 4 eggs per tip^21^; hence, *G. pecorum* is more likely to contaminate the whole pasture. *G. pecorum*’s oviposition strategies including not chasing the host, ovipositing a large number of eggs, and scattering oviposition are important for its adaptation to the arid desert steppe and facilitate its infection of Przewalski’s horse.

Host plant volatiles may induce gravid insects to land^28^. The species and quantities of volatiles released by a host plant are influenced by a variety of factors, such as the species, tissue, and organ, physiological state, phenological state, circadian rhythm, and environment^17,29–33^. In this study, 35 volatiles were identified from GP1, I-L, and II-D, 36 from II-L/GP2, 37 from III-L, 40 from GP3, and 27 from II-T. Insects can detect or identify only a small portion of the volatiles released by their host plants. For example, Bruce and Pickett^20^ reported that in a complex food-source plant, volatile mixtures typically include 3–10 candidate key compounds for host recognition.

The relative contents of alcohols were significantly higher in II-L than in I-L or III-L, but significantly lower in II-T than in II-L or II-D, and in GP3 than in GP1 or GP2. Furthermore, 3-hexen-1-ol,(*Z*)- was one of two volatiles common to all samples, and its relative content was highest in II-L and lowest in II-T. Additionally, 3-hexen-1-ol,(*Z*)- is strongly attractive for adult *Agrilus planipennis* Fairmaire, 1888 (Coleoptera: Buprestidae) and has been used in trapping pests and monitoring population dynamics in forests^34,35^. Cui et al.^36^ reported that the relative content of 3-hexen-1-ol,(*Z*)- was high in leaves of *Malus halliana* Koehne, 1890 and *Malus domestica* Borkh., 1803, the host plants for adult *Agrilus mali* Matsumura, 1924 (Coleoptera: Buprestidae). Also, the combination of 3-hexen-1-ol,(*Z*)- with egg-yellow and light-green sticky trap plates reportedly enhances attraction of *A. mali*^37^*.* Finally, 3-hexen-1-ol,(*Z*)- is also a plant lure used by *Batocera horsfieldi* Hope, 1839 (Coleoptera: Cerambycidae) and *Anoplophora chinensis* Forster, 1771 (Coleoptera: Cerambycidae)^38^.

The relative contents of esters were significantly lower in II-L than in I-L or III-L, and in II-L than in II-D or II-T, but they were significantly higher in GP3 than in GP1 or GP2. Among them, 3-hexen-1-ol,acetate,(*Z*)-, which was unique to I-L, II-D, and GP3, was one of the top five. The attractant effect of purple prism traps with 3-hexen-1-ol,(*Z*)- for adult *A. planipennis* was greater than those with unbaited controls, but addition of 3-hexen-1-ol,acetate,(*Z*)- did not yield a synergistic effect^35^. However, 3-hexen-1-ol,(*Z*)- and 3-hexen-1-ol,acetate,(*Z*)- are synthesized by fatty acid derivatization^39^, and 3-hexen-1-ol,(*Z*)- can be esterified by an alcohol acyltransferase to 3-hexen-1-ol,acetate,(*Z*)-^40–42^. In this study, the low relative content of esters in II-L was caused by the absence of 3-hexen-1-ol,acetate,(*Z*)-, possibly as a result of inhibition of alcohol acyltransferase activity.

Caprolactam was one of the two common volatiles in the top five of all samples, and its relative contents were highest in II-T and lowest in III-L. Caprolactam is the most abundant compound in the host plants of *Apolygus lucorum* Meyer-Dür., 1843 (Hemiptera: Miridae), such as *Glycine max* (L.) Merr., 1917, *Gossypium* spp., *Medicago sativa* L., 1753, *Vigna radiata* (L.) Wilczek, 1954, and *Capsicum frutescens* L., 1753^43^, and its relative content is highest in roots of *Triticum aestivum* L., 1753, the host plant of *Melanotus cribricollis* Faldermann, 1835 (Coleoptera: Elateridae)^44^. Caprolactam is also the main volatile in *Eurohypnum leptothollum* (C. Muell.) Ando., 1966, the host plant of *Kaburagia rhusicola* Takagi, 1937 (Hemiptera: Pemphigidae)^45^, and in the hedgerow plant *Ilex crenata* var. *convexa* (Makino) Rehder, 1949^46^*.*

The relative contents of aldehydes were similar in all samples. Among them, 3-hexenal, one of the top five aldehydes, was detected in all samples except II-L and II-T. Hexanal, which was detected only in II-L, III-L, and GP1, was also one of the top five, and this compound elicits a strong electroantennogram response by both male and female adult *Protaetia brevitarsis* Lewis, 1879 (Coleoptera: Scarabaeidae)^47^. Hexanal is a strong attractant for *Sphaerophoria menthastri* Linnaeus, 1758 (Diptera: Syrphidae)*, Plutella xylostella* Linnaeus, 1758 (Lepidoptera: Plutellidae), and *Chrysopa septempunctata* Wesmael, 1841 (Neuroptera: Chrysopidae)^48,49^, and its content is positively correlated with the number of Locustoidea insects^50^.

Species-specific^51^ and ratio-specific^52^ odor recognition are the means by which phytophagous insects recognize host plants. The selection ratio of male *G. pecorum* to *S. caucasica* in different growth periods was basically similar, but its female tended to select *S. caucasica* at early and flourishing periods and the ratio to select the latter is the highest among the three growth periods. The ratio of females preference for *S. caucasica* over *C. latens* was higher, whereas males’ selection of the two plant species was similar (unpublished data). Further studies are needed to clarify whether the preference of female *G. pecorum* for GP1 and GP2 is a result of specific components or ratio-specific components. Our findings will enable *G. pecorum* electroantennogram response studies and the development of *G. pecorum* attractants, which could reduce the incidence of severe infection by *G. pecorum* of Przewalski’s horse. Moreover, such attractants could enable wild release, rather than the current artificial semi-wild release, of Przewalski’s horse.

## Materials and methods

### Study area

The Kalamaili Nature Reserve (KNR) (44°36′–46°00′ N, 88°30′–90°03′ E), dominated by the Gobi Desert and semi-desert landscape, is located in the northeast of Jungar Basin, Xinjiang, People’s Republic of China. It is dry and windy with little rain in spring, and hot and dry during the short summer. The annual precipitation in KNR is 159.1 mm, typical of a temperate continental arid climate^53^. Chenopodiaceae, Cruciferae, Asteraceae, Leguminosae, and Poaceae are the dominant plant species in KNR, accounting for more than 60% of the total species^54^. Many rare wild animal species are found in KNR, such as *Equus przewalskii*, *Equus hemionus*, *Ovis ammon*, and *Gazella subgutturosa*^53^.

### Experimental design

Due to special life cycle and biological characteristics of *G. pecorum*, it is difficult to define the oviposition period: the mature larvae of *G. pecorum* are discharged from the body with equine feces and then pupate into the ground and become adults. Adults of the fly need to mate and lay eggs immediately, because their life span is very short, only 1 to 4 days. When larvae of the fly are collected in the field, the relatively concentrated time when larvae are discharged from the body is judged according to the number of larvae, which is the peak of larvae discharge, and external environment temperature during the period is recorded. In this work, based on the mature larvae status of *G. pecorum* collected in the field in 2019, including the peak of larvae discharge, the local ambient temperature, the prediction formula for the pupa development history of the fly^55^, and its adult life span, and by monitoring the emergence of larvae via field culture, the oviposition phenological stages of the fly in 2019 was defined as follows: oviposition (stage II), 7 days, May 17–23; preoviposition (stage I), 15 days before stage II; and postoviposition (stage III), 15 days after stage II.

From April 24 to June 7, 2019, under sunny and windless weather conditions, the following fresh plants were collected at 11:00–12:00 am (Beijing time) in the winter–early spring captive areas and late spring–summer free-foraging sites of Przewalski’s horses and in the vicinity of water sources and donkey roads: (1) *S. caucasica* shoots during the three oviposition phenological stages of *G*. *pecorum* (preoviposition [I-L], oviposition [II-L], and postoviposition [III-L]); (2) shoots of three plant species during the oviposition stage (*S. caucasica* [II-L], *S. orientalis* [II-D], and *C. latens* [II-T]); and (3) *S. caucasica* shoots from three different growth periods (early growth period [GP1], flourishing growth period [GP2; *i.e.*, II-L], and late growth period [GP3; *i.e.*, heading period]). A total of seven samples, each with three duplicates, were collected for further experiments.

### Volatile collection

For each duplicate of a sample, 100.00 g of fresh plant shoots were weighed and placed in an oven bag (Reynolds, Richmond, VA) to collect volatiles. The plant materials were dried at 65℃ for 48 h to determine their dry weight after the collection of volatiles was completed.

Before volatile collection, the air in the oven bag removed out using the QC-1S air sampler (Beijing Municipal Institute of Labour Protection, Beijing, China). The oven bag was refilled with air filtered through the activated carbon of a drying tower, which was connected through polytetrafluoroethylene tubes to an air sampler and an activated absorption pipe (CAMSCO, Houston, TX) filled with Tenax TA (60/80 mesh; Alltech, Deerfield, IL) to form a closed system. The air was recycled to collect volatiles for 5 h at a flow rate of 1 L/min. After one sampling, the absorption pipe was sealed and kept at − 20℃ until gas chromatography-mass spectrometry (GC–MS) analysis.

### Automatic thermal desorption gas chromatography–mass spectrometry analysis

Volatiles collected in the absorption pipes were enriched using a Turbo Matrix 650 Automatic Thermal Desorber (PerkinElmer, Waltham, MA) with a two-stage heating program. The carrier was high-purity helium. The volatiles in the adsorption pipes were desorbed at 260℃ for 10 min, and then reabsorbed in the cold trap (− 30℃), which was heated to 300℃ at a rate of 40℃/s for 5 min, and finally moved into the GC through a capillary transfer line (250℃). The conditions of the Clarus 600 Gas Chromatograph (PerkinElmer) were as follows: DB-5MS UI chromatographic column (30 m • 0.25 mm • 0.25 μm; Agilent Technologies, Santa Clara, CA); initial temperature of 40℃ for 2 min, increased to 180℃ at 6℃/min, followed by an increase to 270℃ at 15℃/min for 3 min. The conditions of the Clarus 600 T Mass Spectrometer (PerkinElmer) were as follows: electron ionization at 70 eV; mass scan range of 30 to 500 m/z; and interface and ion source temperatures of 250℃ and 230℃, respectively.

The volatiles were analyzed by TurboMass 5.4.2 GC–MS software (PerkinElmer, Shelton, CT). The volatiles were identified by matching their retention times, characteristic ions, and mass spectra with the NIST 08 library (National Institute of Standards and Technology, Gaithersburg, MD). The relative contents of individual volatiles were calculated by the area normalization method.

### Data analysis

Data analysis was performed using SigmaPlot Version 12.5 and SPSS Version 22.0 software. The normality of the distribution and the homogeneity of the data were examined by the Shapiro–Wilk test and Levene’s test, respectively. One-way analysis of variance (ANOVA) or the *t*-test was used for quantitative data. Nonparametric testing was used for data that did not meet the requirements for normality and homogeneity after transformation. All statistical tests were performed at a 5% significance level, and data are expressed as mean ± standard error (SE).
